# Finite helical axis for the analysis of joint kinematics: comparison of an electromagnetic and an optical motion capture system

**DOI:** 10.1186/s40945-015-0008-7

**Published:** 2015-08-25

**Authors:** Corrado Cescon, Andrea Tettamanti, Marco Barbero, Roberto Gatti

**Affiliations:** 1 Rehabilitation Research Laboratory, Department of Business Economics, Health and Social Care, University of Applied Sciences and Arts of Southern Switzerland (SUPSI), Manno, Switzerland; 2 Laboratory of Analysis and Rehabilitation of Motor Function, Neuroscience Division, San Raffaele Hospital, Milan, Italy

**Keywords:** Finite helical axis, Motion capture, Kinematics, Electromagnetic sensors, Optoelectronic systems

## Abstract

**Background:**

The analysis of joints kinematics is important in clinical practice and in research. Nowadays it is possible to evaluate the mobility of joints in vivo with different motion capture techniques available in the market. Optical systems use infrared cameras and reflective markers to evaluate body movements, while other systems use electromagnetic fields to detect position and orientation of sensors. The aim of this study was the evaluation of two motion capture systems based on different technologies (optical and electromagnetic) by comparing the distribution of finite helical axis (FHA) of rotation during controlled rotations of an object in different positions.

**Methods:**

The distribution of position and angle errors of the FHA were extracted by optical and electromagnetic system recordings during a controlled rotation of a low friction stool in different positions in a controlled environment.

**Results:**

The optical motion capture system showed lower angle and position errors in the distribution of FHA while the electromagnetic system had higher errors that increased with increasing distance from the antenna.

**Conclusions:**

The optical system showed lower errors in the estimation of FHA that could make it preferable with respect to electromagnetic systems during joint kinematics.

## Background

The investigation of joints kinematics is important for clinical practice and in research in order to quantify the joint impairments and to suggest possible interventions. In some cases like the zygapophysial joints of the cervical spine, the analysis of kinematics is difficult because of the anatomical morphology and of its multi-structural composition. Nowadays it is possible to evaluate the mobility of joints in vivo with different motion capture techniques. Among different parameters used for the analysis of joint movements, such as range of motion, angular velocity and jerkiness, Woltring introduced the use of instantaneous helical axis (IHA) [1] of rotation of a body segment with respect to the other. When the movements are analyzed in discrete steps, the axis of rotation between two time instants is defined the finite helical axis (FHA) [1]. Position and orientation in space of the FHA can be defined by a number of parameters, and the descriptive statistics of these parameters has been recently used to express the stability of the motion in cervical spine and in knee and ankle joint analysis [2–6]. In a recent work we proposed a method to quantify the distribution in space of the FHA during cervical movements [7].

Different motion capture systems are available in the market and their characteristics and specifications are described by the manufacturers in the data sheet, but the limitations of each technology are often difficult to evaluate. Some researchers analyzed the behavior of FHA in relation to measurement errors [1, 7], but, although indications of precision and accuracy of each system are indicated in the technical manuals, there is still unclear the behavior of different motion capture systems in the extraction of FHA. Some systems use infrared cameras and reflective markers to evaluate body movements (optical systems), but they are sensitive to light sources and the markers can be hidden due to shadow effects [8]. Inertial systems do not provide information on the position of the body segments, which can be only estimated with double integrations that suffer from drift effects [9]. Electromagnetic systems have high resolution in terms of position and orientation but they suffer from field distortions, especially when there are metal objects in the proximity of the electromagnetic antenna [10].

In many application fields, like videogames, virtual or augmented reality, the field distortion effects are not a major limitation. Indeed in such applications, the aim is real time visual feedback to accomplish a specific task. On the other hand, for joint kinematic analysis in rehabilitation, the precision of the measurements may become a critical feature as the aim is to detected differences from normative data or among populations.

Finally, the accuracy of motion capture system can be tested by measuring the actual position and orientation of sensors with respect to the reference frame and is often reported in the user manuals.

When analyzing joint kinematics, and in particular cervical kinematics, the subject is usually standing or sitting and is moving only the head with respect to the trunk, in this way the movements induce small displacement and an optimal combination of precision and “local” accuracy of the system could provide reliable estimates of FHA. In this context an ideal hinge with a unique and invariant axis of rotation is an important requirement in order to evaluate a motion capture system in computing the FHA.

The aim of this study was the evaluation of two motion capture systems based on different technologies (optical and electromagnetic) by comparing the distribution of finite helical axis (FHA) of rotation during controlled rotations of an object in different positions.

## Methods

### Procedure

**Fig. 1:**
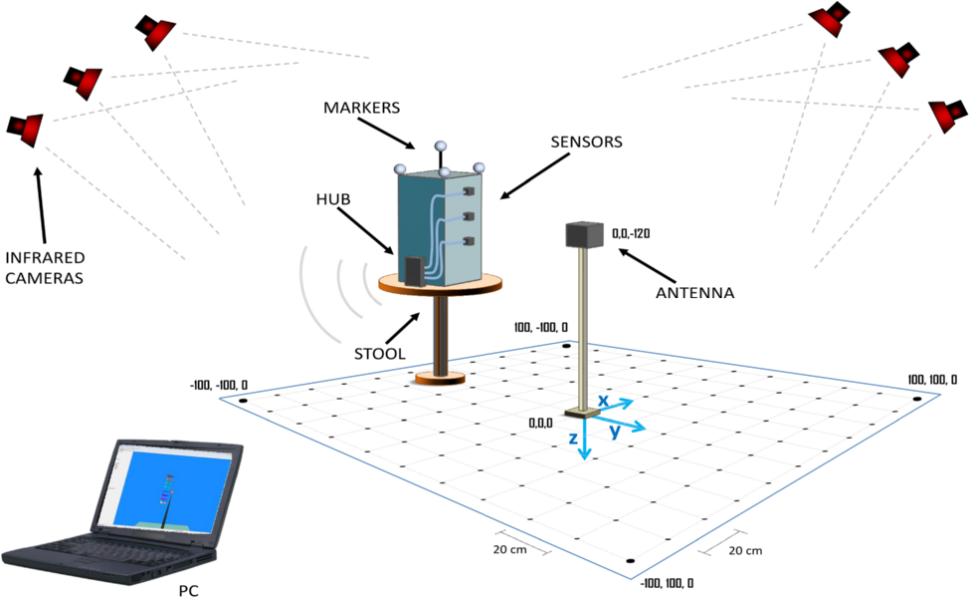
Graphical representation of the experimental set-up. The antenna and the rotating box are shown as well as the grid where the box was positioned during each acquisition

A square grid of 121 equally spaced points (11 x 11, distance between adjacent points: 20 cm) was drawn on the floor of the laboratory (see Fig 1). A low friction rotating stool with a plastic box was positioned over the grid with five reflective markers on its top, and three electromagnetic sensors were fixed with adhesive tape on one of the sides of the box along a vertical line, with 15 cm spacing in between (see Fig. 1). The antenna of the electromagnetic system was positioned on a wooden stick in the center of the grid, at a height of 120 cm from the floor. The base of the stool was moved in each of the points of the grid. The minimum distance of the stool from the antenna was set to 40 cm to allow the rotation of the stool, thus the points in the central square of the grid (3 x 3) were not used. For each position of the grid (11 x 11 points minus 3 x 3 points: 112 total points) the stool was manually turned around its rotation axis from the neutral position clockwise of approximately 270° and backwards until the neutral position two times. The angular velocity was approximately 120°/s in order to avoid vibrations or artifacts due to the inertia of the box. For each of the 112 positions, a recording was performed with both electromagnetic and optical motion capture systems.

### Optical system

The optoelectronic position data were recorded with the BTS Elite (BTS, Italy) motion capture system, which included 6 infrared cameras positioned in standard points of the measurement room (four in the upper corners and two in the top center of the longest walls). The system was previously calibrated using a standard stick with four markers. The room had rectangular shape of 12 × 8 m and had no metal object or electromagnetic sources nearby. Four reflecting markers were positioned on the top of the plastic box, three in the corners of the top surface of the box (a rectangle of 25 × 30 cm) and the fourth in its center, at the top of a plastic stick of 20 cm, in order to have a minimal configuration of non-coplanar markers. The location of the optical markers was chosen in order to have the 4 markers always visible by all cameras, and their location on the object was similar to the position of markers on the head during cervical kinematics usually performed in clinical studies. The axis of rotation was along the z direction, perpendicular to the floor (see Fig. 1). The optical data from the infrared cameras were transformed by the Elite system which provided the positions in space (x, y, z) of the four markers positioned on the box at a sampling rate of 100 Hz.

### Electromagnetic system

The electromagnetic data were recorded with the Polhemus-G4 acquisition system (Polhemus, USA), a device that tracks position and orientation of sensors relative to a source in three dimensions. The system has been used and shown to be accurate to within ± 0.2° [11, 12]. In our test, three sensors were fixed to the rigid plastic box at three different heights from the floor (105, 120, and 135 cm) at a distance of 15 cm from the rotation axis (see Fig. 1). The location of the sensors with respect to the antenna was selected in order to be similar to what is usually performed in clinical practice during cervical kinematics, where the sensors are positioned on the subject’s forehead. The wires were secured with adhesive tape to prevent traction on the sensors. The electromagnetic source was positioned over a wooden stick positioned in the middle of the room at a height of 120 cm from the floor (coordinates 0, 0,+120). The sensors were attached to a transmitter (hub) which had a wireless connection to a laptop PC, and continually recorded the position and orientation (x, y, z, pitch, roll, yaw) for each of the three sensors at a sampling rate of 120 Hz. Each marker was considered individually.

### Signal processing

The position data obtained from the Elite system were analyzed in order to obtain the position and direction cosine matrices for each time instant using the singular value decomposition (SVD) technique. The Euler angles from the Polhemus-G4 system were converted into direction cosine matrices. The box was assumed to be a rigid body and its center was assumed to be in the center of the box at a height of 120 cm.

For both systems the position and orientation of the box were used to compute the Finite Helical Axis (FHA) for each time instant. The algorithm used for the extraction of FHA was previously described [7, 13]. For each acquisition one FHA was extracted for each pair of time instants selected in order to have always the same angular distance in between. The angle between the two frames was set to 10° according to the results of a previous study [7].

**Fig. 2:**
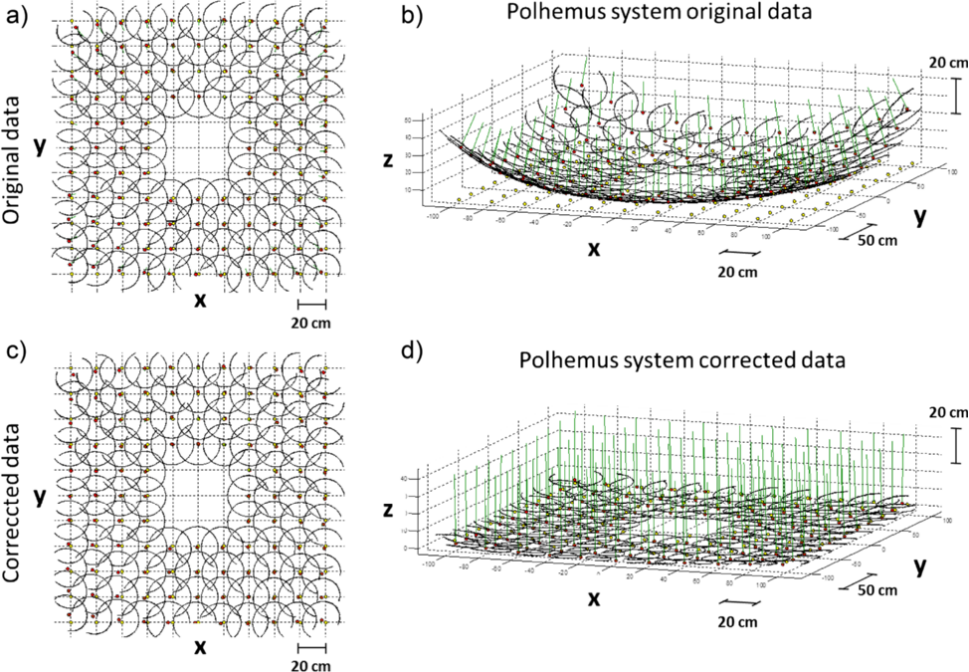
Compensation of the position data error due to the distortion of the electromagnetic field. The top panels **a**, **b** show the original coordinates of the trajectories of the second Polhemus sensor during the different acquisitions in top and side view respectively. The bottom panels **c**, **d** show the same data after the application of the bidimensional function of the second order that minimizes the distortion in the z axis

Since the electromagnetic data were affected by static field distortions, position data were compensated with a bi-dimensional function of the second order, whose parameter were empirically identified in order to reduce the deviation of the z positions from the theoretical horizontal plane (Fig. 2).

Since the recordings were lasting approximately 10 s, the number of FHA detected for each acquisition was about 1200 for the electromagnetic system and 1000 for the optical system. The intersections of each of the FHA with the horizontal plane at the height of the antenna were identified, and the distances between each FHA from the average intersection point was defined as position error. In addition the angle between each FHA with respect to the vertical was defined as angle error.

### Statistical analysis

The mean value and standard deviation of the distribution of position and angle errors were computed for each position of the box and stool on the grid. The distribution of angle and position errors extracted with the two detection systems were compared using the 2-way Analysis of Variance (ANOVA). The fixed factors were: type of sensors (Elite, Polhemus sensor 1, Polhemus sensor 2, Polhemus sensor 3), and stool location on the grid as fixed factors. Post hoc differences were investigated using the Wilcoxon rank sum test for equal medians. Shapiro-Wilk test was applied to signals to evaluate the data normality. Significance level was set to α = 0.05.

## Results

**Fig. 3:**
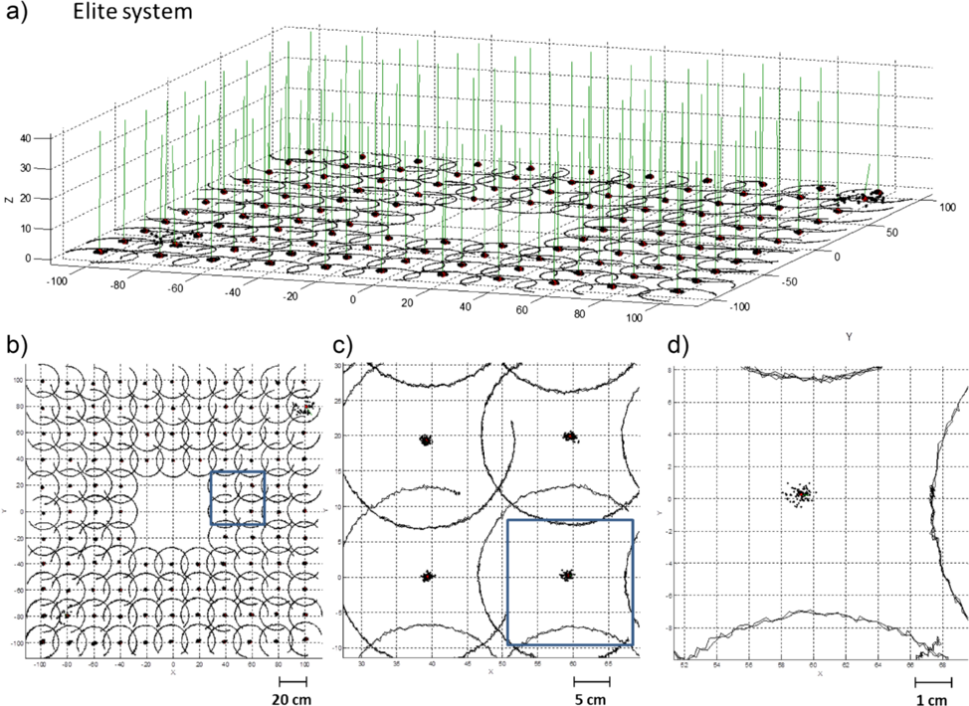
Representation of the trajectory of one virtual point of the rigid body obtained by the analysis of the positions of the Elite markers and the FHA relative to each of the acquisitions. **a** side view **b** top view, **c** zoomed view **d** detail of the distribution of intersection points of the FHA with the horizontal plane

**Fig. 4:**
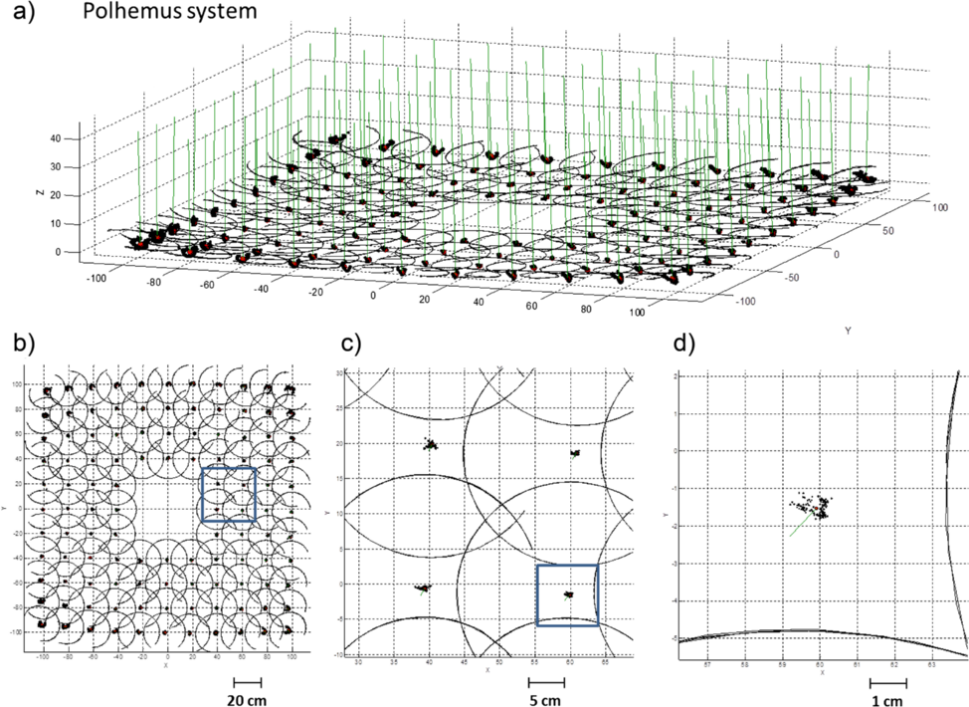
Representation of the trajectory of the second Polhemus sensor and the FHA relative to each of the acquisitions. **a** side view **b** top view, **c** zoomed view **d** detail of the distribution of intersection points of the FHA with the horizontal plane

Figure 3 and 4 shows the trajectories of one virtual point of the rigid body obtained by the analysis of the positions of the Elite markers during each rotation of the box positioned over the 112 points of the grid. It is possible to see the distribution of intersection points of the FHA with the horizontal plane.

Figure 4 shows the same trajectories of Fig. 3, but with position and orientation data acquired with the second of the Polhemus sensors (120 cm from the floor).

**Fig. 5:**
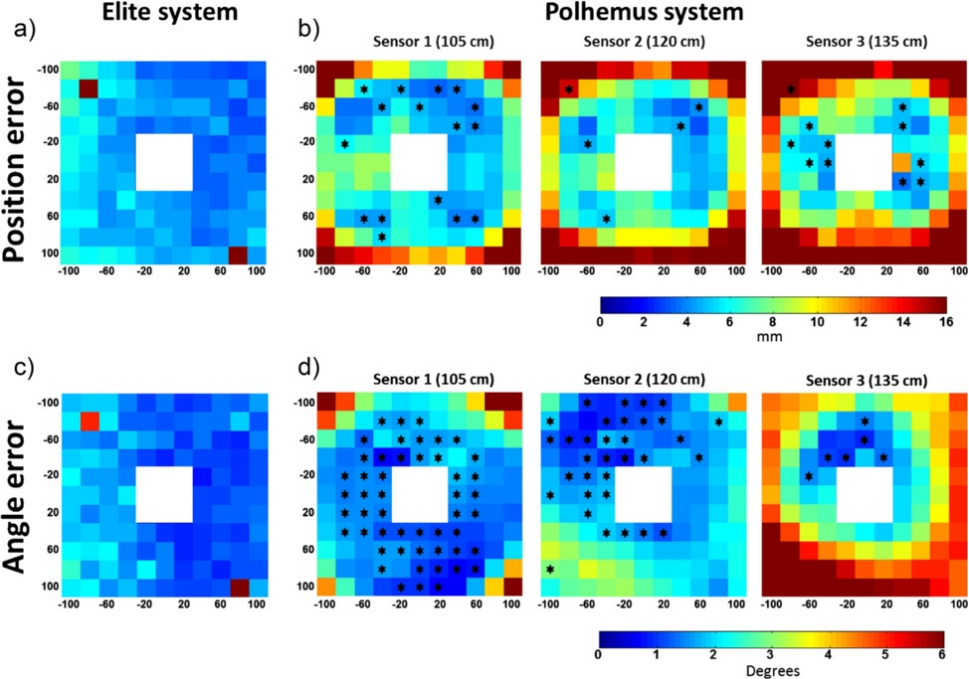
Graphical representation of the mean values of the distribution of position errors **a** and **b** and angle errors (**c** and **d**) of each of the FHA with respect to the mean FHA for each of the positions of the stool in the horizontal grid. The mean errors of the Elite system for both variables are shown on panels a and c, while panels b and d show the same results for the three sensors of the Polhemus system. Values are expressed in mm and degrees respectively. Areas with no differences between each polhemus sensor with respect to the Elite are indicated by stars

Figure 5 shows the distribution of the distances of each FHA from the average FHA for each acquisition. Each “pixel” represents one position of the stool over the grid, and the color represents the position error in mm and angle error in degrees respectively. Stars indicate the results of the post-hoc comparison, and show which locations of the grid of the Polhemus sensors have no statistical difference in FHA dispersion with respect to the Elite system.

The 2-way ANOVA showed a significant difference (*p* < .01) for position and angle errors was observed between the Elite system and each of the three sensors of the Polhemus systems in each of the positions of the stool, with higher errors observed in the Polhemus system. Figure 5 and Table 1 summarize the results of the statistical analysis.

**Table 1:** Summary of the two-way ANOVA analysis, the F and P values of the differences of position and angle errors between Polhemus and Elite systems

	Sensor 1 (105 cm)		Sensor 2 (120 cm)		Sensor 3 (135 cm)	
	F	*P*	F	*P*	F	*P*
Position error	8472	*P* < <0.01	12699	*P* < <0.01	63236	*P* < <0.01
Angle error	11579	*P* < <0.01	43338	*P* < <0.01	97544	*P* < <0.01

## Discussion

The main result was the increase of angle and position error with increasing distance from the antenna in the Polhemus system. Since the field generated by the antenna is spherical, the resolution was indeed expected to decrease with increasing distance from the antenna. At a distance of 40–60 cm in any direction in the xy plane, the position error is about 5 mm, while increasing the distance up to 80 cm the error increases to 10 mm or more. The variability of resolution with the distance from the antenna makes the electromagnetic sensor difficult to use in applications where the movements of the sensors are larger than 40–50 cm. On the other hand the optical systems with infrared cameras are based on different points of view, thus reducing the distance of the sensors from a camera will increase their distance from other cameras, allowing an almost constant resolution of the system in large portions of the measurement room.

Anyway even at distances below 60 cm from the antenna, the position error of the Polhemus system is above 5 mm while for the Elite system the error is about 4 mm and constant in almost all the investigated grid of positions.

The angle error of Polhemus system shows an asymmetric behavior, with larger angle errors (about 3 or more degrees) in the lower left corner of the grid (coordinates −100,100,0) suggesting that the field deformations are not perfectly spherical, and thus are harder to compensate. The angle errors of Elite systems are almost constant and below 1.5° in all the positions on the grid.

From Figs. 3 and 4 it is possible to observe that the positions from the electromagnetic system (Polhemus) are smoother than the positions extracted from the optical system. This phenomenon is because the resolution of the optical systems depends only from the number of pixels of the cameras and their magnifying lenses, while the electromagnetic sensors have unpredictable distortions and there is no easy way of compensating the distortions. On the other hand the optical systems have the limitation of sunlight and unwanted reflections but for joint measurements seem to be more reliable and easy to use. In addition the absence of cables on the subject reduces the mechanical interferences that could reduce the range and the smoothness of movements of the subject.

The optical system seems to be preferable for the analysis of joint kinematics. Anyway, the clinical relevance of the observed differences in precision between the two systems, may be not relevant in comparing different populations. Further studies are required to address this specific issue.

### Limitations

The main limitation of this study is the choice of the two systems. The market of optical motion capture sensors is growing fast and in the recent years different products have been released with higher resolution and image quality besides more advanced algorithms for the reconstruction of marker position, while our Elite system was developed about 10 years ago. In addition the number of cameras could be increased, the number of markers on the box could be increased, and the distance between the cameras and the stool could be reduced, thus the error in the estimation of the position of FHAs could be dramatically reduced.

Similarly, the electromagnetic system is 5 years old, and there are other systems available on the market that include more powerful antennas, with presumably lower distortion effects.

Another limitation is the use of a bi-dimensional second order function for the compensation of the field distortions. The parameters of the function were empirically selected in order to minimize the static position errors of the sensors. In Fig. 2 it is possible to notice that at the corners of the grid there is a residual distortion effect. A higher order function or an analytical approach for understanding the reasons for such distortion could have improved the compensation of the distortion. Anyway for our purpose, the optimal compensation was not mandatory, since the sensors were moving in a small portion of the grid, where the distortion was negligible.

Another way to reduce distortion could be the use of multiple antennas configuration, which was not investigated in the present study.

Moreover, the experimental procedure involved rotations around only one axis, and the stool was moved on the same plane. The number of acquisition could be increased, but we had to fix some parameter in order to limit the amount of collected data. Lastly the stool is not an ideal hinge and could have some intrinsic error, but we put a big attention in gently rotating the stool trying to minimize any artifact due to the manual movements.

## Conclusion

Two different detection systems (optical and electromagnetic) were compared in a controlled environment and the optical systems was observed to have lower position and angle. The electromagnetic system was affected by field distortions, and had position and orientation errors that strongly depended on the distance from the antenna. The optical motion capture technologies seem to be preferable for the analysis of joint kinematics.
